# Neurotrauma and Inflammation: CNS and PNS Responses

**DOI:** 10.1155/2015/251204

**Published:** 2015-03-31

**Authors:** Bruno Siqueira Mietto, Klauss Mostacada, Ana Maria Blanco Martinez

**Affiliations:** Laboratório de Neurodegeneração e Reparo, Departamento de Patologia, Faculdade de Medicina, Hospital Universitário Clementino Fraga Filho, Universidade Federal do Rio de Janeiro, 21941-550 Rio de Janeiro, RJ, Brazil

## Abstract

Traumatic injury to the central nervous system (CNS) or the peripheral nervous system (PNS) triggers a cascade of events which culminate in a robust inflammatory reaction. The role played by inflammation in the course of degeneration and regeneration is not completely elucidated. While, in peripheral nerves, the inflammatory response is assumed to be essential for normal progression of Wallerian degeneration and regeneration, CNS trauma inflammation is often associated with poor recovery. In this review, we discuss key mechanisms that trigger the inflammatory reaction after nervous system trauma, emphasizing how inflammations in both CNS and PNS differ from each other, in terms of magnitude, cell types involved, and effector molecules. Knowledge of the precise mechanisms that elicit and maintain inflammation after CNS and PNS tissue trauma and their effect on axon degeneration and regeneration is crucial for the identification of possible pharmacological drugs that can positively affect the tissue regenerative capacity.

## 1. Introduction

Wallerian degeneration (WD) is a multicomplex phenomenon occurring in the distal portion of injured nerves [1–3]. The term was established in tribute to Augustus Waller's seminal observations on how distal nerves change their morphology after being cut [4]. Although originally described in axotomized peripheral nerves, WD also occurs in both central and peripheral axons separated from their parental cell body [5]. WD is known to be triggered not only by a traumatic insult, but also in several neurodegenerative diseases (amyotrophic lateral sclerosis, Alzheimer's disease, and Parkinson's disease), in which affected axons share pathological signs with what is normally observed in axons undergoing traumatic injury-induced WD [6]. More importantly, WD also includes a multitude of changes in nonneuronal cells (i.e., glial cells, fibroblasts, and immune-derived cells) that, together, strongly influence the patterning of axon degeneration and regrowth [7–9]. This scenario is evident when we compare WD occurring in central tracts with peripheral axons; central nervous system (CNS) injury has been associated with an impaired regenerative process, along with a marked deficiency in WD progression [5]. After an insult to the CNS structure, neurons, glial, endothelial, and meningeal cells are mechanically and/or physiologically destroyed, triggering the molecular signals that lead to an amplification of the primary insult [10–13]. Concomitantly with the primary events, the secondary wave of insults is generated in a response to the breakdown of the blood-brain barrier (BBB) and activation of glial cells, leading to changes in the CNS microenvironment and eliciting a robust inflammatory response [14, 15]. Conversely, injured peripheral nerves are often associated with a certain degree of regeneration [16, 17], and the baseline reason for this success is generally associated with activation of Schwann cells and macrophages, along with the inflammatory reaction elicited in injured nerves [18–20]. Although inflammation in the peripheral nervous system (PNS) has been linked to successful axon regeneration, a complete understanding of how this immune reaction (i.e., pro- and anti-inflammatory cytokines) modulates degeneration and regeneration responses is not yet completely clarified. In addition, the precise role of inflammation after CNS trauma is still a matter of intense debate. Several reports have demonstrated that inflammation is detrimental to CNS neurons [21–23], while other studies have shown the opposite, that is, a positive role of inflammation in promoting CNS regeneration [24, 25]. In this review, we will focus on how WD-derived inflammation impacts CNS and PNS responses upon injury, particularly on the dual sides of the inflammatory reaction and glial and immune-cell activation in the two systems.

## 2. Spinal Cord Pathology after a Traumatic Injury

**Figure 1:**
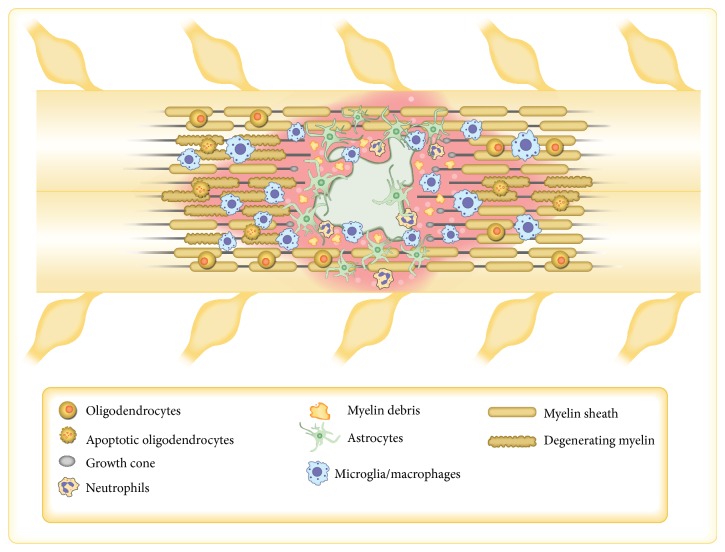
Schematic representation of spinal cord injury site. Insult to the spinal cord immediately generates a robust hemorrhagic area followed by glial cell activation. At the same time, axons undergoing degeneration and dead neuronal cell bodies elicit the recruitment of inflammatory cells from the periphery. Soon after injury, neutrophils reach the tissue parenchyma and begin to secrete molecules that can exacerbate tissue and vascular damage. Over the time course of the injury, monocytes infiltrate the spinal cord, where they transform into macrophages, persisting from days to months at the injury site and thus contributing either to the degenerative or to the regenerative process. Spared neurons start the regenerative machinery, which fails to cross the injury site formed by the glial scar.

The pathophysiology of spinal cord injury (SCI) consists of a primary event that causes neural cell death and interruption of axonal connections. The distal stump of the long axons undergoes a degenerative process called WD, while the proximal stump retracts, making the surviving cell bodies more vulnerable to subsequent events (Figure 1) [26]. Within a few hours after SCI, a secondary cascade of events takes place, which is characterized by interruption of the normal blood flow followed by hemorrhage, edema, inflammation, release of extracellular matrix molecules and pro- and anti-regenerative factors, and activation of the cell death machinery [27]. In contrast to the PNS, the adult mammalian CNS has a limited regenerative capacity, which has been related to changes that occur in its microenvironment. Hemorrhage, elicited by the mechanical impact, leads to a progressive hemorrhagic necrosis that affects tissue not primarily affected by the trauma itself. Studies addressed to prevent hemorrhage spreading, through pharmacological treatment with glibenclamide, an FDA-approved antidiabetic drug, found that the animals that received this drug showed a reduction in the hemorrhagic area, accompanied by preservation of the locomotor ability [28, 29]. These findings can be attributed to the effect that glibenclamide exerts on the endothelial cell surface receptor SUR1 (sulfonylurea receptor 1) by inhibiting its function and thus preventing capillary fragmentation, with consequent reduction in the lesion volume and better preservation of intact fibers [28]. Another important hallmark of SCI is the presence in the parenchyma of the injured tissue of inhibitory molecules, such as chondroitin sulfate proteoglycans (CPSGs), which are released from glial cells (mainly astrocytes), forming a physical and chemical barrier called the glial scar, which functions to prevent the regenerative axons from crossing the injury site [30, 31]. Other types of inhibitory molecules, such as Nogo-A, derived from oligodendrocytes, are highly expressed after injury and suppress the capacity of the growth cone to elongate [30]. Furthermore, myelin components such as myelin-associated protein (MAG), oligodendrocyte myelin protein (OMGp), semaphorin 4D (SEMA 4D–CD100), and ephrin (B3) also act as inhibitory molecules for CNS regeneration [30–33]. In contrast, proregenerative molecules (i.e., NGF, TGF-*β*, PDGF, EGF, BDNF, and oncomodulin) are secreted by neural and inflammatory cells in order to promote axon elongation [25, 34, 35]. The effectiveness of the neurotrophic factors depends on the balance between pro- and antiregenerative molecules in a stoichiometric fashion. Therapies based on delivery or increase of neurotrophic factors have been extensively employed and showed an improved recovery of locomotor function [36]. Concomitantly with these events, activated glial cells recruit blood cells to the injury site, exacerbating tissue inflammation.

## 3. Inflammatory Cell Response after SCI

Contrary to previous belief, the CNS does not constitute an immunoprivileged system, since it shares many commonalities with other systems. Therefore, the immune response that follows a SCI has received increasing attention in recent decades, and it is becoming clear that inflammation exerts an important effect on the progress of degeneration and regeneration following a traumatic lesion [14]. Immediately after SCI, the BBB breakdown and blood-vessel fragmentation lead to an increased expression of leukocyte adhesion molecules on the surface of the endothelial cells and an overflow of plasma proteins to the parenchyma of the injured tissue, including pro- and anti-inflammatory cytokines [11, 12]. At the same time, microglial cells become activated and act by eliminating cells and extracellular matrix debris, as well as by releasing molecular mediators of inflammation, and recruitment and activation of cells [37]. These early inflammatory events recruit leukocytes from the peripheral blood in a time-dependent manner.

## 4. Participation of Neutrophils in SCI

Following an injury to the CNS, neutrophils can reach the injured parenchyma by activated endothelial cell signaling and/or by secreted molecules derived from microglia [38, 39]. Microglia activation and secretion of neutrophil chemoattractant molecules can be induced by damage-associated molecule patterns (DAMPs) or alarmins, including hyaluronan, heparin sulfate, heat-shock proteins, necrotic cells, ATP, nuclear factors, galectins, IL-1*α*, IL-33, uric acid, thioredoxin, and high mobility group box 1, which are present in the lesioned tissue milieu [40–42].

Neutrophils are the first cell line to respond to tissue damage. In mechanical injuries, neutrophils are recruited within a few hours after tissue damage, peaking at 1 to 3 days post-injury (dpi). The bactericidal function of these cells is well described in infections [40], but the role of these cells in CNS trauma is not yet completely elucidated. Neutrophils are recruited in sequential steps from the vasculature to the “inflamed site.” These steps start with a contact between neutrophils and the endothelial wall, followed by rolling and arrest steps, before the transmigration into the tissue parenchyma [41]. Under different stimuli, endothelial cells increase the expression of E- and P-selectins, while neutrophils express their ligands L-, E-, and P-selectins. Then, neutrophils are arrested at specific sites by activation of *β*2 integrins (LFA-1, Mac-1) that interact with ICAM-1, expressed by endothelial cells, through chemokine receptors. Once neutrophils are arrested on the endothelial surface, they can transmigrate to the tissue through the endothelial cell junctions by interacting with PECAM-1, ICAM-1, VE-cadherin, JAMs, and CD99 [41].

When activated, neutrophils secrete large amounts of proteolytic enzymes such as elastase and metalloproteinases (MMPs). These enzymes are able to act in different extracellular matrix substrates and the endothelial cells, damaging the endothelium and thus facilitating leukocyte migration through the injured tissue [10, 43, 44]. In CNS, neutrophils have been extensively linked to deleterious effects. This has been associated with the release of proinflammatory cytokines such as interleukin- (IL-) 1*β*, IL-6, and tumor necrosis factor alpha (TNF-*α*) and chemokines such as macrophage inflammatory protein-1 (MIP-1), macrophage chemoattractant protein-1 (MCP-1), and IL-8 [45]. By inhibiting neutrophil migration using anti-P-selectin antibody in a spinal cord injury model, Taoka and collaborators [21] found a reduced accumulation of neutrophils at the injury site, which was closely related to a decrease in the hemorrhagic area, and sparing of the neurological function. In another study, Stirling and collaborators [46] depleted neutrophils after a contusive spinal cord injury using anti-Ly6G/GR-1 antibody. In contrast to the findings of Taoka and coauthors [21], neutrophil depletion by using anti-Ly6G/GR-1 antibody worsened the wound healing and functional outcome [46]. Stirling and coauthors [46] also observed that the mice that received anti-Ly6G/GR-1 showed an increase in the expression of macrophage chemoattractants (MIP1*γ*/CCL9, KC/Gro-*α*/CXCL1, G-CSF, and MCP1/CCL2), which suggests an attempt to compensate for the lack of neutrophils by increasing the recruitment of macrophages to the injury site. Despite the elevated expression of the macrophage chemoattractants, the treatment did not increase the number of microglia/macrophages within the injured spinal cord [46]. Interestingly, as the expression of macrophage chemoattractants increased, the expression of wound healing molecules decreased at 48 h after injury in anti-Ly6G/GR-1 treated mice. Also, the depletion of neutrophils decreased the GFAP levels, thus strongly suggesting a reduction in the wound healing and scar formation. Concomitantly with these findings, the treated mice showed a poorer functional recovery, which was related to less preserved white matter and axons in the injury site [46].

Inflammation induced in nerves has been shown to increase the nerve regenerative capacity by enabling axons to grow and sprout collateral branches in response to neurotrophins; this is clear in the context of the optic nerve injury model. Oncomodulin (Ocm), a small Ca^2+^-binding protein, acts as a neurotrophin and participates in inflammation-induced regeneration [25, 47]. After injection of zymosan into the retina of the mouse optic nerve, there is an increase in macrophage recruitment, followed by enhancement of Ocm expression and secretion, improving axon elongation [25, 47]. In the beginning it was believed that macrophages were the main source of Ocm, but recent data suggest that neutrophils are the major source of Ocm production and secretion in the first 24 h after intravitreal injection of zymosan. Ocm concentration starts to decrease at about 72 h after injury, a time when the number of neutrophils decreases and macrophage numbers start to increase [48]. In spite of this proregenerative function that has been attributed to neutrophils in the optic nerve injury model, its role in SCI is still under debate and needs further elucidation.

## 5. The Role Played by Microglia/Macrophages in SCI

Microglia are SNC resident glial cells that have several distinct receptors such as cytokines and chemokines on their cell surface, which enable them to recognize different stimuli such as abnormal or unusual concentrations of molecules (for review, refer to [37]). Any disturbance in the CNS environment can turn microglia into an activated state, changing their morphology from a ramified to a round/amoeboid shape. Macrophages originating from the peripheral-blood monocytes are attracted to the lesion site and become indistinguishable from microglia. In the bloodstream, monocytes can be segregated into two distinct subpopulations, based on distinct chemokine receptor and specific surface molecules [49]. The phenotypes Ly6C^hi^/CX3CR1^low^ and Ly6C^low^/CX3CR1^hi^ refer to pro- and anti-inflammatory monocytes, respectively. These cells respond to a large variety of chemokines such as MCP1 (also known as CCL2) and MCP3 (also known as CCL7) and share the same surface receptor CCR2, inducing Ly6C^hi^ recruitment, while Ly6C^low^ responds to CX3CL1, also known as fractalkine [50]. In a sterile inflammation, monocytes are recruited to the inflamed area by interacting with P- and E-selectin, rolling on the blood-vessel walls, using VCAM-1 for firm adhesion and transmigration into the inflamed tissue, where they turn into macrophages [50]. Macrophages have been extensively studied in different models of nervous system disorders, but their different roles are still under debate. Their functions are typically related to phagocytosis of dead cells and tissue debris and to secretion of pro- and anti-inflammatory molecules and neurotrophic factors. They can either exacerbate the injury or promote repair, based on the signals present at the injury site [51]. Macrophages, microglia, and other neural cells secrete high levels of TNF-*α*, which in turn activates a macrophage/microglia program inducing the release of molecules that kill neurons and oligodendrocytes, exacerbating tissue damage. These effects can be attenuated by administration of TNFR1, which sequesters TNF-*α*, reducing its availability to bind to TNF receptors [52]. Furthermore, activated macrophages secrete large amounts of reactive oxygen species and proteases, contributing to further damage of the otherwise spared tissue around the lesion epicenter [22]. In a classical report, Popovich and collaborators [22] depleted the peripheral macrophage population by using systemic administration of clodronate liposome in rats. This treatment reduced macrophage infiltration at the injury site, and this was accompanied by an improvement of the animal's locomotor function, preservation of myelinated fibers, decrease in the cavitation area, and enhancement of axon regeneration.

Studies unrelated to the nervous system revealed that macrophages could be polarized into pro- and anti-inflammatory phenotypes based on the specific pathway that is activated. It is well established that T helper 1 cytokine (Th1) interferon-*γ* (IFN-*γ*) activates macrophages and induces the production of proinflammatory cytokines (IL-12, IL-23, IL-1*β*, and TNF-*α*) and cytotoxic mediators, while T helper 2 cytokine IL-4 drives the macrophage polarization to an anti-inflammatory phenotype by inhibiting the production of proinflammatory cytokines (TNF-*α*, IL-1*β*, IL-2, IL-8, IL-12, and CXCL10), increasing MHCII, and reducing the respiratory burst [53]. These findings led to the concept of “classically” activated macrophages (M1), responsible for IFN-*γ* and TLR signaling, and “alternatively” activated macrophages (M2), responsible for IL-4 and IL-13 signaling. The markers CD16, CD32, CD86, MHCII, and iNOS refer to M1 macrophages (proinflammatory), while Arginase-1 (mouse), CD163, CD204, CD206, YM1, and Fizz1 refer to M2 macrophages (anti-inflammatory) [53]. After a traumatic injury, it is difficult to determine which population of macrophages is present in the tissue, based on the complexity of multiple damage factors that drive the macrophage polarization.

After SCI, both populations are present in the injured tissue parenchyma from 3 to 7 days after injury [51]. However, the signal to sustain an M2 polarization decreases over time, and the M1-polarized macrophages predominate at the injured cord [51]. These cells can persist at the injury site for weeks, exerting their neurotoxic effects and impairing the tissue regenerative capacity. On the other hand, M2 macrophages are not neurotoxic, and these cells are able to promote long-distance axon growth [51]. Attempts to modulate macrophage polarization to the M2 phenotype have yielded positive results in animal models, by decreasing the lesion length and promoting a better functional recovery after SCI, indicating a promising therapeutic strategy [49].

After SCI, there is a marked increase in the expression of CX3CR1 by macrophages. Importantly, the deficiency of this receptor improved neurological recovery and tissue sparing after SCI by modulating the microglia/macrophage phenotype, diminishing their neurotoxic effects [54]. Of interest, the lack of CX3CR1 on the macrophage surface exacerbates neuronal loss in models of Parkinson's disease and amyotrophic lateral sclerosis [55].

As already mentioned, Yin and collaborators [25] showed that increasing macrophage recruitment by zymosan injection after optic nerve crush also increases the secretion of Ocm, a neurotrophic factor, resulting in the promotion of axon regrowth through the injury site. Recently, using conditional phosphatase and tensin homolog (PTEN) knockout mice plus a combination of multiple injections of zymosan and CPT-cAMP (cAMP analogue), which facilitates the binding of Ocm to its receptor, the same group observed that retinal ganglion cells were able to regenerate their axons through distal targets of the visual pathway [47, 56].

## 6. Peripheral Nerve Pathology after a Traumatic Injury

Normally, uninjured peripheral nerves are composed of resident macrophages, fibroblasts, and Schwann cells, the PNS-wrapping glia. Taking into consideration all nucleated nonneuronal cells that are present in a naïve tissue, Schwann cells outnumber resident macrophages by about 10 times [57, 58]. More importantly, by being in close contact with axons, Schwann cells are likely to be the front-line population to react after axon injury. Even in the intact peripheral nerve, Schwann cells constitutively express mRNA for TNF-*α* and IL-1*α*, although only TNF-*α* proteins are found in detectable levels in the uninjured nerve [59, 60]. Conversely, as soon as the nerve is damaged, Schwann cells promptly overexpress a broad panel of inflammatory mediators including TNF-*α*, IL-1*α*, IL-1*β*, MCP-1, MIP-1, IL-10, TGF-*β*, and galectin-3 in a time-dependent manner [2, 7, 8, 60–62], which switch on the inflammatory response. Of interest, this abrupt rise in inflammation (i.e., ~5 to 24 hours after trauma) occurs well before any structural changes are observed in distally severed axons. What are the underlying mechanisms initiating this early inflammatory burst? When the nerve is physically damaged (i.e., transection, crush, and ligation), the injury itself destroys both local cells and surrounding tissue. Consequently, the lesion site will be filled with harmful DAMPS [63, 64]. Therefore, by being in intimate contact with axons, Schwann cells might detect these small changes in nerve homeostasis and hence set a compensatory reaction (i.e., inflammation) in motion [65]. Schwann cells are able to act as antigen-presenting cells through the class I and II major histocompatibility complex (MHC) [66, 67] and mainly because they are adopted with several toll-like receptor (TLR) members, including TLR-2/-3/-4 [68]. Although TLRs are classically related to pathogen recognition, they can also be activated by endogenous sterile molecules, which are broadly produced during nerve degeneration, as mentioned above [69]. For example, after being exposed to necrotic cells, Schwann cells upregulate inflammatory-related genes via TLR-2 and TLR-3 signaling [70]. Also, several proinflammatory cytokines such as IL-1*β* and TNF-*α* are produced after TLRs-NFKb activation [71]. These findings support the notion that Schwann cell upregulation of genes associated with inflammation might be due to activation of TLRs by nerve-derived ligands [64]. A direct* in vivo* functional role of TLRs was elegantly demonstrated by Boivin et al. Taking advantage of TLR-2 and TLR-4 knockout mice, they observed impaired WD and axon regeneration in these deficient animals after sciatic nerve injury [72]. On the other hand, a simple intraneural injection of TLRs ligands in WT-injured nerves augmented macrophage influx and myelin clearance and enhanced motor recovery. Although TLRs signaling affects myelin phagocytosis after nerve injury, it is not clear if degenerating myelin is in fact a TLR ligand or if myelin clearance is an indirect effect resulting from prior TLR activation in the cells.

During the first week after nerve injury, these proinflammatory signals trigger tissue destruction, increase Schwann cell numbers, activate resident nonneuronal cells to produce higher quantities of inflammatory mediators, and recruit circulating leukocytes to degenerated nerves [7, 9]. This feedback loop of nerve fragmentation, cell proliferation, immune cell influx, and proinflammatory cytokine release keeps the nerve inflamed for long periods after injury. One particular cytokine with key effects during this initial stage is the proinflammatory cytokine IL-1*β*. After nerve injury, IL-1*β* reaches maximum levels at 24 hours [20, 60] and is followed by a later and second peak at day 14 [62]. During this initial phase, IL-1*β* induces myelin collapse through a complex cascade involving phospholipase A2 (PLA2) and lysophosphatidylcholine (LPC) activation in Schwann cells [73, 74]. Moreover, expression of PLA2 was observed in distal nerve segments for up to 2 weeks after injury [74]. It has been shown that PLA2 triggers myelin breakdown after hydrolyzing the lipid membrane phosphatidylcholine, which is found in high levels in the myelin sheath, resulting in the generation of large amounts of LPC, a molecule with a natural myelinolytic action [75]. Several other cytokines such as TNF-*α*, IL-1*α*, and MCP-1 can also increase PLA2 expression in both Schwann cells and macrophages, emphasizing the notion that all compact myelin that surrounds severed axons will be fully fragmented into ovoids by, at least, a sustained expression of PLA2-related factors (for review refer to [75]). In addition, PLA2 proteins appear to influence other key aspects of WD in injured nerves. The lack of intracellular PLA2 disturbs myelin breakdown, macrophage recruitment, and myelin clearance, leading to delayed WD and impaired axon regeneration after sciatic nerve crush [76]. Another evidence of myelin breakdown after injury was reported by Jung and coauthors [77], who demonstrated that changes in actin polymerization inside Schmidt-Lanterman incisures are crucial to trigger myelin fragmentation.

Nerve injury-induced inflammation is a precisely orchestrated multicomplex reaction that involves a multitude of inflammatory mediators and cells [7, 8, 78]. In the days following nerve injury, the distal stump undergoes structural changes, leading to its total disintegration [79, 80]. Although injured axons trigger intrinsic self-destruction pathways [81, 82], there is no doubt that cytokines/chemokines and inflammatory cells enhance this fragmentation process. Interestingly, sciatic nerve segments obtained 24 hours after injury, at distances of 10–15 mm from the lesion site, contained high levels of mRNA for several inflammatory mediators, with levels comparable to what is found in injured segments extracted from the lesion site [62]. How can Schwann cells located far from the injury site react with such magnitude if no morphological alterations have already begun at that distance? Physiologically, axons require a constant supply of NMNAT2, an endogenous survival factor produced in the neuron cell body and delivered to axons by anterograde axonal transport [83]. When the nerve is damaged, NMNAT2 transport to distal axons is interrupted and those NMNAT2 still remaining in the distal axons will be rapidly degraded.* In vitro* experiments suggest that the half-life of NMNAT2 is less than 4 hours in transected neurites [83]. Therefore, it might be possible that Schwann cells are able to “sense” not only alterations in the nerve microenvironment, but also slight disturbances in the levels of critical axonal factors. Whether or not axonal internal molecules are able to signal to Schwann cells to trigger inflammation is a fundamental question that requires further investigation.

## 7. The Role Played by Macrophages during PNS Wallerian Degeneration

**Figure 2:**
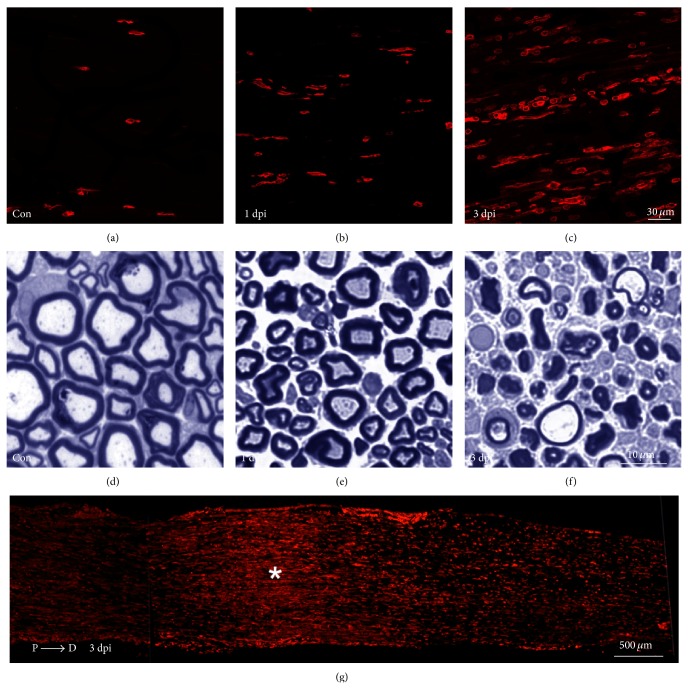
Macrophage accumulation and nerve pathology after PNS trauma. (a), (b), and (c) are representative images of CD11b+ cells (red staining)—which is suggestive of macrophages—in control sciatic nerves, 1 and 3 days post injury (dpi), respectively, at the distal portion of the nerve (~2 mm from the injury site). Nerve integrity ((d), (e), and (f)) is observed at the distal portion of the nerve (~2 mm from the injury site) at the same time points mentioned in (a), (b), and (c), respectively. Image (g) is a longitudinal section of injured sciatic nerve at day 3, showing accumulation of CD11b+ cells (red staining) in the entire nerve and showing augmented CD11b+ numbers at the injury site (asterisk) and distal portions of the nerve. Scale bar: (a), (b), and (c) = 30 *μ*m; (d), (e), and (f) = 10 *μ*m; (g) = 500 *μ*m.

**Figure 3:**
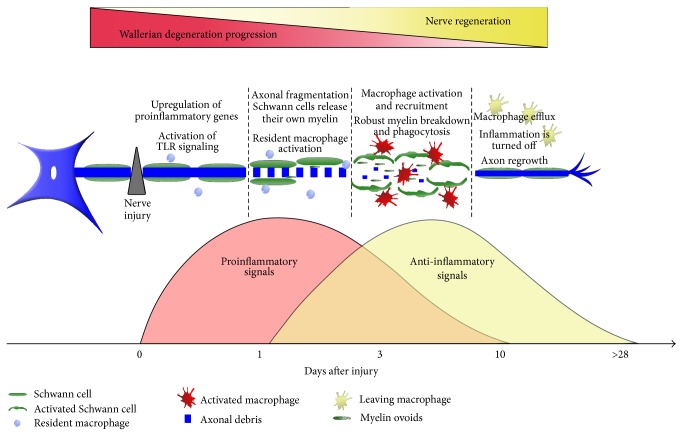
Development of nerve fragmentation-associated events after PNS trauma.

Nerve injury induces recruitment, accumulation, proliferation, and activation of macrophages [7, 9, 84] (Figure 2). Although resident macrophages begin to divide after PNS injury [57, 58], those coming from the periphery supplement the population of these cells in degenerated nerves. Macrophages penetrate the nerve at around days 2 to 3 after injury, as a result of the initial inflammatory wave, and reach maximum levels at days 7–14 [58]. Similarly to chemokines, the humoral system appears to have a potential role in mediating macrophage functions. Vargas and coauthors [85] demonstrated that endogenous antibodies broadly target nerves undergoing WD, and this opsonization is critical to induce macrophage phagocytosis. Since macrophages are professional phagocytes, as soon as they enter the nerve they begin the phagocytosis of cellular debris, which was initially performed by Schwann cells [86, 87]. By doing that, macrophages drastically increase the rate of cellular debris clearance in the injured distal nerve stump. As mentioned, macrophages can be functionally polarized into the M1 or M2 phenotype [53]. Currently, the* in vivo* dynamics of macrophage polarization after nerve injury is still a matter of intense debate. Ydens and colleagues [88] demonstrated that, after mouse nerve axotomy, macrophages acquire only the M2 phenotype. Contrariwise, other studies found M1-type macrophages in injured nerves [89–91]. These divergent findings might be related to different factors, such as type of injury, time points assessed, and mainly the panel of macrophage markers used by the authors. Another important feature is that myelin ingestion by macrophages is involved in their polarization toward the M2 profile [92, 93]. Although M1 and M2 macrophages are well described in* in vitro* procedures, during* in vivo* situations macrophages are exposed to all types of stimuli that are disease/injury- and tissue-specific, probably leading to complex phenotypes and mixed populations of cells [53]. Therefore, since macrophages are a dynamic heterogeneous population, it would be more relevant to address the question of what biological functions are performed by these cells after PNS injury than to determine the M1/M2 ratio. Collectively, the inflammatory nature of WD is a well-regulated and precisely timed phenomenon shaped by multiple factors (i.e., M1/M2 macrophages, pro- and anti-inflammatory cytokines) that together orchestrate the evolution of nerve pathology (Figure 3). Knowledge of the macrophage repertoire and functions after nerve injury has advanced our understanding of how inflammation is controlled after PNS injury. At present, the majority of studies have looked at the mechanisms that attract macrophages to degenerated nerves. Interestingly, the issue of how macrophages exit injured nerves has been little investigated. One study from the Laboratory of Samuel David elegantly addressed this issue and demonstrated that macrophage clearance from injured sciatic nerves is mediated via repulsive interactions between Nogo receptors in macrophages and their ligands, which are present in remyelinated axons [94]. Several anti-inflammatory cytokines, such as IL-10, are widely produced after nerve injury, helping to keep the inflammation under control and switching off the inflammatory reaction. For example, around day 7 after injury, IL-10 is highly expressed in damaged nerves, and this upregulation is associated with reduced levels of GM-CSF, a powerful leukocyte chemoattractant [61]. In addition, important intracellular inflammatory molecules, such as SOCS1 and SOCS3, have been characterized as being involved in the regulation of inflammation during nerve degeneration and regeneration [95].

Inflammation is a prerequisite for successful nerve regeneration in the PNS, as it serves, for example, to eradicate harmful myelin from the nerve microenvironment. However, coordination of pro- and anti-inflammatory signals during WD is crucial and must be tightly controlled to ensure successful axon regeneration.

## 8. Galectin-3 and PNS Injury

Of particular interest to our laboratory is the role played by galectin-3 after PNS injury [19, 20]. Original studies in WT and WLDs (slow Wallerian degeneration) injured mice revealed that, after sciatic nerve injury in WT animals, activated Schwann cells and macrophages efficiently engulf myelin after galectin-3 activation [96]. Conversely, injured sciatic nerves from WLDs mutant mice, which display a marked reduction in the development of WD with no morphological signs of degeneration up to 14 days after injury [97], do not express galectin-3 in Schwann cells and macrophages, emphasizing the notion that these cells only upregulate galectin-3 after being exposed to myelin and axonal debris [98]. In fact, galectin-3 might favor myelin phagocytosis by exerting its effects both inside and outside the cells [8, 98]. Based on these observations and taking the advantage of using specific galectin-3 knockout (Gal-3 ko) mice, our group hypothesized that sciatic nerve regeneration after sciatic nerve crush would be impaired in these knockout animals, due to inefficient myelin phagocytosis. To our surprise, we in fact observed acceleration in nerve regeneration in the Gal-3 ko mice [19], which was accompanied by an increased number of Schwann cells and macrophages. In order to explore the underlying reasons related to this enhancement in nerve regeneration, we next aimed to determine the pattern of myelin breakdown and phagocytosis in mice lacking galectin-3. Also, we questioned whether or not the inflammatory reaction is altered in these Gal-3 ko mice after nerve injury, since galectin-3 mediates cytokine production [99–101]. We observed a higher expression for both mRNA and protein levels, for two important proinflammatory molecules (IL-1*β* and TNF-*α*) in the Gal-3 ko injured nerves [20]. We also found increased numbers of cells expressing TLR-2 and TLR-4 at 4 days after trauma [20]. In accordance with our data, peritoneal macrophages obtained from Gal-3 ko showed upregulated levels of mRNA for TLR-2 and IL-1b [99]. Moreover, galectin-3 was found to be necessary for polarization of macrophages into the M2 phenotype [101], although no study has assessed the M1/M2 ratio after peripheral nerve injury in Gal-3 ko mice. Finally, we also observed that the absence of galectin-3 in Schwann cells and macrophages increased their* in vitro* phagocytic activity, compared to their WT counterparts [20]. Although galectin-3 has been associated with efficient phagocytosis [96, 98], Gal-3 ko mice might develop compensatory mechanisms to increase the phagocytic potential, such as alterations in TLR-2/-4 signaling [20]. Galectin-3 is a pleiotropic protein with multiple effects, and this is related, at least in part, to its cellular location and target signaling [102]. For example, it has been shown that galectin-3 inhibits* in vitro* proliferation of Schwann cells [103] and increases the number of fibroblasts [104]. The precise contribution of galectin-3 to PNS degeneration and regeneration still needs elucidation and is a promising field for further exploration.

## 9. How Does Inflammation Prepare the Tissue for Newly Growing Axons?

After a traumatic lesion, the axon becomes separated into two segments: a proximal segment that remains in contact with the cell soma and a distal segment that becomes separated from the neuron cell body. The distal nerve stump undergoes a cascade of events called WD [4, 105], while the proximal stump begins to be prepared for axon regeneration. WD is initiated within 24 to 48 h by the entry of calcium into the axoplasm, leading to activation of proteases, such as calpains, that promote axoplasm disintegration [106, 107]. Soon after this, macrophages are attracted to the site of injury and, together with SC, initiate intense phagocytosis and removal of the degenerating axon and myelin debris. Almost immediately after injury, Schwann cells in the distal stump of the nerve begin the process of differentiation and modify their gene expression [108] by decreasing myelin protein expression and start to express genes related to regeneration, such as c-Jun and growth-associated protein 43 (GAP-43), neurotrophic factors, neuregulins, and their receptors [17]. In addition, Schwann cells start to proliferate and migrate to form specialized cellular columns, referred to as Bands of Büngner, that act as a guide pathway for growing axons [17]. Inside these bands, Schwann cells support the growth potential of injured neurons by releasing basal lamina components such as laminin and type IV collagen [109–114]. When SC contacts the regenerating axons, the process of remyelination is started [115].

While the distal stump axon disintegrates and provides a permissive microenvironment for regeneration, it also generates signals that target the neuronal cell body, resulting in its change from transmitting to a growth-promoting phenotype. These changes reflect variations in the metabolic activity of neurons, which, as a result, start to produce substances that are important for axonal elongation, initiating the process of axon regeneration from the proximal stump [116]. But, how does inflammation affect the process of axon regeneration? In the injured peripheral nerve, the proinflammatory reaction accelerates the disintegration of nerve fibers but primarily prepares nonneuronal cells (i.e., glial and immune cells) and the distal stump microenvironment to receive newly growing axons. This scenario is best exemplified by the impaired axon regeneration when WD is disturbed [117–120] or when the macrophage response is deficient [18, 72, 85]. Conversely, augmented inflammation seems to improve axon regrowth, not only in injured peripheral nerves [19, 20] but also in the optic nerve, where regeneration barely occurs after trauma [24, 56, 121]. First, inflammation recruits circulating macrophages to efficiently phagocytize damaged myelin, which contains several axonal-growth inhibitors [122–124]. In addition to being involved in myelin clearance, macrophages are important sources of several factors related to promoting axonal regeneration, such as Ocm. They also release large quantities of IL-1*β*, and its production regulates the generation of nerve-growth factor (NGF) by fibroblasts and Schwann cells [125]. Another important aspect is the role of LIF (leukemia inhibitory factor) for neural regeneration [17]. After injury, LIF is retrogradely transported toward the cell body and induces the expression of regeneration-associated genes such as the activating transcription factor-3 [126] and growth-associated proteins [127, 128], among others [129]. Indeed, mice lacking LIF showed deficient peripheral nerve regeneration after lesion [130]. Although WD-derived inflammation is associated with several beneficial effects for axon elongation, the shutdown of this inflammatory process is also essential for nerve regeneration. Uncontrolled inflammation is the underlying reason for innumerable nerve pathologies, including neuropathic pain [131] and autoimmune diseases, such as Guillain-Barré syndrome [132].

## 10. Concluding Remarks

**Figure 4:**
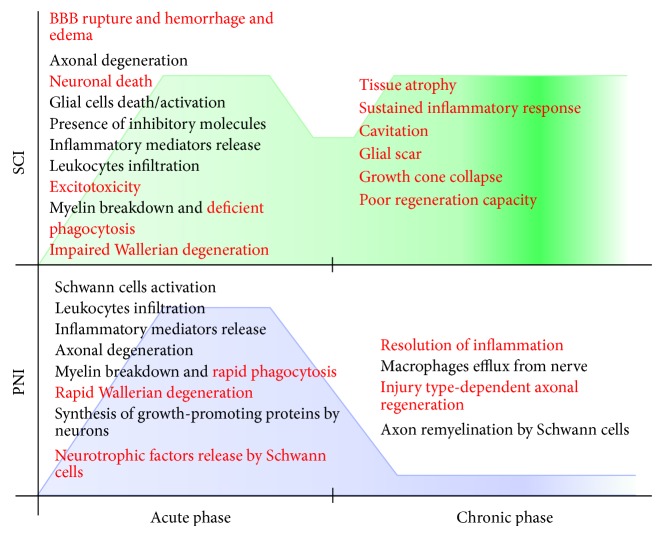
Commonalities (in black) and differences (in red) between SCI and peripheral nerve injury (PNI). Soon after a traumatic injury, both spinal cord and peripheral nerves elicit a rapid and robust inflammatory response (acute phase), whereas at later time points (chronic phase) SCI presents a second wave of inflammatory cells recruitment, which is detrimental to axon regeneration. Conversely, in peripheral nerves there is a marked resolution of the inflammatory response, which is correlated with successful regenerative process. Green and blue backgrounds represent the inflammatory curves after SCI and PNI, respectively.

Nowadays it seems clear that the CNS and PNS respond differently to traumatic injuries (Figure 4). For example, several axonal-growth inhibitory molecules present in degenerated CNS myelin are not properly removed from the axonal microenvironment [5, 123]. Conversely, PNS myelin debris is efficiently cleared from the nerve milieu after trauma, creating a permissive environment for axon regrowth [5, 87]. Moreover, while Schwann cells in the PNS differentiate and assume major roles that support axonal elongation, glial cells in the CNS do not efficiently aid axon regrowth [31, 114, 133]. One of the key features of WD in the PNS is the robust accumulation of recruited macrophages along the entire distal portion of the nerve, which differs from what is seen after spinal cord injury, where macrophage influx occurs mainly at the lesion site. These immune cells may favor axon regeneration in different ways but mainly by phagocytosis of degenerated myelin and axon debris. However, during CNS inflammation, macrophage responses have been correlated with detrimental effects, such as increase of the secondary damage events [30, 51]. Contrary to this view, the Benowitz Research Group has demonstrated that CNS-derived inflammation can induce axonal regeneration in the injured optic nerve [27, 47, 48, 56]. These findings suggest that CNS and PNS regenerating abilities depend on several factors (i.e., glial cells, immune cells, effector molecules, time elapsed after injury, and lesion magnitude, among others). The combination and balance of these different factors will lead to either abortive or successful axon regeneration. In order to achieve the optimum benefit from the beneficial side of inflammation after nervous system injury, it is necessary to fully understand how resident glial cells and immune cells crosstalk and behave upon traumatic injury to the nervous system.
